# A case report of mevalonate kinase deficiency in a 14-month-old female with fevers and lower extremity weakness

**DOI:** 10.1186/s12887-019-1617-1

**Published:** 2019-07-20

**Authors:** Tiziana Coppola, Bradford Becken, Heather Van Mater, Marie Theresa McDonald, Gabriela Maradiaga Panayotti

**Affiliations:** Duke Children’s Primary Care, 4020 North Roxboro Street, Durham, NC 27704 USA

**Keywords:** Periodic fever syndrome, Mevalonate kinase deficiency, HyperIgD syndrome, Mevalonic aciduria, Inflammation, Arthralgias, Rash

## Abstract

**Background:**

This case follows a 14-month-old female, who despite multiple presentations to several physicians, continued to have recurrent febrile episodes with gross motor delay. Her case revealed an often missed diagnosis of Mevalonate Kinase Deficiency, that now has an FDA approved treatment that both reduces recurrence and produces remission.

**Case presentation:**

A 14-month-old female with a history of gross motor delay, frequent Upper Respiratory Tract infections, and otitis media presented to an urgent care for inconsolability and refusal to bear weight on her right leg. She had recently been treated with amoxicillin for acute otitis media and had developed a diffuse maculopapular rash, without any associated respiratory or gastrointestinal distress that persisted beyond cessation of the antibiotics. The patient presented multiple times to an urgent care over the subsequent week for fussiness, fever, anorexia, lymphadenopathy, with labs concerning for worsening anemia and elevated inflammatory markers. Subsequently, the patient was admitted to the hospital for suspected osteomyelitis versus oncologic process. X-Ray imaging of the patient’s lower extremities showed osseous abnormalities inconsistent with infection. A metabolic work-up showed elevated urine mevalonic acid, and follow-up genetic testing was positive for mutations in both copies of her mevalonate kinase gene. This led to the diagnosis of MKD.

**Conclusions:**

Often, episodic presentations require multiple perspectives to reveal the underlying cause. This case illustrates how apparent simple febrile episodes has the potential for more complexity upon further evaluation.

## Background

Mevalonate Kinase Deficiency (MKD) is a rare autosomal recessive disorder due to a defect in cholesterol and isoprenoid synthesis. The mutation in mevalonate kinase leads to reduced enzymatic activity or absent activity in cholesterol synthesis. MKD can be further broken down into two syndromes, Hyperimmunoglobulin D Syndrome (HIDS) and Mevalonic Aciduria (MVA), based on the residual amount of enzyme activity of mevalonate kinase [1].

The hallmark signs of the disease are episodic periods of fevers, adenopathy, arthralgias, abdominal pain, and rash with elevated inflammatory markers. Fevers occur intermittently, often with no signs of an infection or clear trigger. Attacks can also be brought on by an illness or vaccination. Patients characteristically have elevated urine mevalonic acid and can also have elevated IgD paraprotein. MKD is considered a periodic fever syndrome, similar to Familial Mediterranean Fever (FMF) or TNF-receptor associated periodic syndrome (TRAPS). This disease is often misdiagnosed due to its rarity. Currently, there is no cure for MKD. Treatment with monoclonal antibodies targeting cytokines are highly effective, reducing frequency and severity of attacks, and producing long term remission in many [1]. Bone marrow transplantation has also been investigated as a treatment in severe cases [2].

## Case presentation

A 14-month-old female with a history of gross motor delay and iron deficiency anemia presented to an urgent care with right hip pain. On evaluation, she had cervical and inguinal lymphadenopathy, an elevated ESR (109), CRP (10.19), anemia (Hgb 7.6) and WBC of 10 with 14% bands. Bilateral hip X-ray showed persistent undertubulation without fracture. After having several similar presentations for fever and inconsolability with significantly elevated inflammatory markers and concerning x-rays, she was admitted to the hospital for further evaluation. Intake exam was notable for hepatosplenomegaly, central hypotonia and erythematous maculopapular rash. The differential diagnosis included storage disorders, metabolic disorders, and infiltrative processes, such as leukemia.

Brain MRI was unrevealing, only noting cervical lymphadenopathy. Echocardiogram was unremarkable. Blood smear did not show leukemic blasts. Her rash was consistent with scabies and treated with permethrin. Her extensive metabolic work-up was normal (including urine mucopolysaccharides, creatine kinase, acylcarnitine profile, and plasma amino acids), except for elevated urine mevalonic acid. Repeat urine organic acids performed during another febrile episode again showed elevation of urine mevalonic acid. Genetic testing revealed two mutations in her mevalonate kinase gene with one allele having deletion of exons 10–11, while the other allele having a variant of p.Asn166Lys of unknown significance. Given mutations on both alleles for her mevalonate kinase enzyme, she was diagnosed with MKD.

Our patient is now 23 months old and thriving. Since her diagnosis of MKD, she was started on Anakinra with an excellent response, but she developed diarrhea and was transitioned to Canakinumab. To date, she has had no recurrence of fevers, rash, hepatosplenomegaly, or arthralgias. She is receiving physical and occupational therapy for her gross motor delay, and her skills have improved significantly. She is now walking without difficulty.

## Discussion & Conclusions

MKD is an autosomal recessive disease caused by mutations in the enzyme mevalonate kinase. Mevalonate kinase is an enzyme required for both cholesterol synthesis and the production of non-sterol isoprenoids, which are utilized throughout the cell in physiological processes, such as in the electron transport chain. Mutations in mevalonate kinase produce a spectrum of disease, from a more mild disease known as HIDS to a more severe form, known as MVA. The spectrum of disease is based on the residual enzyme activity remaining. For patients with HIDS, the enzymatic activity ranges from 1 to 20%, whereas in MVA, the enzymatic activity is less than 0.5% [1].

Incidence of this disease is hard to estimate, as there is lack of genetic screening, and patients are not automatically registered into an international database. The highest incidence of known MKD is in the Netherlands, where the original gene mutation (p.Val377Ile) of mevalonate kinase was first discovered. Most patients with MKD are heterozygotes with missense mutations. Due to its high incidence, screening for HIDS is included in the periodic fever work-up in the Netherlands. As of 2007, the international HIDS database has only 20 registered US cases [2, 3].

It can take up to 8–10 years from symptom onset to diagnosis. With the predominant symptom being episodes of recurrent fevers, clinicians build a broad differential including recurrent infections, periodic fever syndromes, autoinflammatory conditions, or myelodysplastic conditions [4]. The length of fever episodes, ethnicity and age are similar to that seen in other periodic fever syndromes, such as TRAPS, FMF, Periodic Fever with Aphthous Stomatitis, Pharyngitis and Adenitis (PFAPA), Muckle-Wells syndrome, and Neonatal Onset-Multisystemic Inflammatory Disease. With episodes of fever, arthralgias and rash, patients can be misdiagnosed as having Still’s Disease, also known as systemic-onset Juvenile Rheumatoid Arthritis. However, if the initial presentation is predominantly fevers with abdominal pain, they can be misdiagnosed with Familial Mediterranean Fever. The aphthous ulcers can be mistaken as symptoms of Bechet’s disease or PFAPA [1, 5]. With several other conditions mimicking MKD, diagnosis often remains elusive.

Diagnosis is further complicated by lack of accurate laboratory testing. During an acute attack, patients will have elevated inflammatory markers and may have elevated IgD paraprotein or IgA paraprotein. However, some patients, especially those who are younger than 3 years of age, may not show elevated IgD paraprotein and/or IgA paraprotein [6, 7]. Furthermore, IgD elevation may occur in other autoinflammatory diseases and does not increase the likelihood of finding a mutation in mevalonate kinase enzyme [1]. Urinary mevalonic acid is elevated during attacks in HIDS patients to varying degrees, but in MVA, it is constantly elevated [7]. The urine organic acid analysis may not pick up small elevations in urine mevalonic acid [4]. The reported sensitivity for urine mevalonic acid is high at about 92%, yet it is still unable to identify all patients afflicted by MKD. It can be utilized as a screening modality for suspected MKD patients [2, 3]. Given the variability in lab values, diagnosis must be confirmed with mutation analysis of mevalonate kinase gene or decreased/absent mevalonate kinase enzyme activity [4]. In patients who have recurrent fevers, arthralgias, abdominal pain, aphthous ulcers and normal IgD levels, genetic testing should be sent [1].

While patients with MKD will have elevated urinary mevalonic acid, symptoms from MKD arise from a decrease in non-sterol isoprenoids. The decrease in production of one non-sterol isoprenoid, geranylgeranyl-phosphate, leads to activation of Caspase 1 through Rac 1 signaling. With activation of Caspase 1, IL-1B precursor is converted to its active form as an inflammatory cytokine [3, 8]. The high level of inflammatory cytokine, IL-1B, is further amplified by reduced apoptosis of lymphocytes leading to a heightened immune response [1]. Unlike most inborn errors of metabolism, patients with MKD have an inflammatory phenotype and lack common complications of inborn errors of metabolism, such as hypoglycemia, metabolic acidosis, ketosis or hyperammonemia [4, 6].

Often unprovoked, patients with MKD will have their first fever episode within the first year of life. Episodes can last anywhere from 3 to 7 days, with symptoms including fevers greater than 40 °C, along with vomiting, diarrhea, lymphadenopathy, splenomegaly, arthralgias, myalgias, maculopapular rash, and aphthous ulcers. Patients typically have a prodromal period prior to development of high grade fevers. Patients will subsequently develop pharyngitis, adenopathy and rash. Usually, the most severe symptom is the abdominal pain that can be mistaken as an acute abdomen [1, 3]. The inflammatory attacks for MKD patients decrease as a patient ages. According to the HIDS database, 90% of patients had > 6 febrile attacks in the first decade, while only 73% of patients had the same number of attacks in the second decade of life [8]. Life expectancy is not adversely affected with HIDS [1].

While patients with MVA also have recurrent episodes of fever and associated symptoms, the episodes usually occur more than twice as often when compared to patients with HIDS [2]. They present with febrile episodes in the first few months of life, with the possibility of death in infancy. However, the most distinguishing characteristic separating MVA from HIDS is the level of neurological impairment [3]. With the disruption of cholesterol biosynthesis, neuronal myelination is compromised, thus creating neurologic effects in patients with MVA [9]. MVA patients are born with dysmorphic features including frontal bossing, hypertelorism, and triangle facies. MVA patients can have ocular manifestations, such as uveitis or cataracts. They have failure to thrive, developmental delay, intellectual disability, and seizures [1, 3]. Patients with MVA usually are hypotonic and develop ataxia as they age [3, 9]. HIDS patients can also develop neurological symptoms later in life, such as ataxia and epilepsy, confirming an overlapping spectrum of illness [4]. Long term complications of MKD occur throughout the spectrum of disease and are due to the chronic inflammatory state. Complications include Type AA amyloidosis, joint contractures, abdominal adhesions, and macrophage activation syndrome [2].

Treatment for MKD is supportive. During acute attacks, patients can take NSAIDs or steroids for symptomatic relief. Long-term therapies currently target the inflammatory nature of the illness with the ultimate goal of decreasing pro-inflammatory cytokines. There is one FDA approved medication, Canakinumab, which is an IL-1B receptor antagonist. Canakinumab along with Anakinra, an IL-1 receptor antagonist, have shown varying rates of success in both partial and complete remission of MKD [2, 3, 8, 10–14]. Adalimumab, a TNF-alpha antibody, has shown both partial and complete remission of severe MKD [2, 15]. Alendronate, a bisphosphonate, produced resolution of recurrent fevers for a patient with MKD, in spite of in-vitro studies showing bisphosphonates also inhibiting cholesterol biosynthesis [16, 17]. A trial of simvastatin in six patients with HIDS failed to show a statistically significant difference in number of days with fevers [18]. In severe cases of MKD, bone marrow transplantation has been successfully used, however there are few studies done given the rarity of the disease [19–21].

## Data Availability

Not applicable

## Abbreviations

AA Amyloidosis: Amyloid A Amyloidosis
CRP: C-Reactive Protein
ESR: Erythrocyte Sedimentation Rate
Hgb: Hemoglobin
HIDS: Hyper-immunoglobulin D Syndrome
IL-1B: Interleukin-1B
JIA: Juvenile Idiopathic Arthritis
MKD: Mevalonate Kinase Deficiency
MVA: Mevalonic Aciduria
TNF: Tumor Necrosis Factor
URI: Upper Respiratory Tract Infection
WBC: White Blood Cell
